# Identification of a pyroptosis-immune-related lncRNA signature for prognostic and immune landscape prediction in bladder cancer patients

**DOI:** 10.1007/s12672-024-00998-y

**Published:** 2024-05-02

**Authors:** Fuguang Zhao, Zhibo Jia, Hui Xie

**Affiliations:** 1 Department of Urology, The Third Medical Center, Chinese People’s Liberation Army (PLA) General Hospital, Beijing, 100039 People’s Republic of China; 2 School of Medicine, Hebei North University, Zhangjiakou, 075000 People’s Republic of China; 3 Departments of Urology, the First Affiliated Hospital of Fujian Medical University, Fuzhou, 350005 People’s Republic of China

**Keywords:** Pyroptosis, lncRNA, Lmmune, Bladder cancer

## Abstract

**Purpose:**

Individualized medicine has become increasingly important in bladder cancer treatment, whereas useful biomarkers for prognostic prediction are still lacking. The current study, therefore, constructed a novel risk model based on pyroptosis- and immune-related long noncoding RNAs (Pyro-Imm lncRNAs) to evaluate the potential prognosis of bladder cancer.

**Methods:**

Corresponding data of bladder cancer patients were downloaded from the Cancer Genome Atlas (TCGA) database. The univariate Cox regression analysis, least absolute shrinkage and selection operator (LASSO) regression analysis, and multivariate Cox regression analysis were employed to establish a predictive signature, which was evaluated by receiver operator characteristic (ROC) analysis and Kaplan–Meier analysis. Furthermore, the immune infiltration, immune checkpoints, and responses to chemotherapeutic drugs were analyzed with this model.

**Results:**

Three Pyro-Imm lncRNAs (MAFG-DT, AC024060.1, AC116914.2) were finally identified. Patients in the low-risk group demonstrated a significant survival advantage. The area under the ROC curve (AUC) at 1, 3, and 5 years was 0.694, 0.709, and 0.736 respectively in the entire cohort. KEGG and GO analyses showed that the Wnt pathway plays a crucial role in the high-risk group. The risk score was significantly related to the degree of infiltration of different immune cells, the expression of multiple immune checkpoint genes, and the sensitivity of various chemotherapeutic drugs.

**Conclusion:**

This novel signature provides a theoretical basis for cancer immunology and chemotherapy, which might help develop individualized therapy.

**Supplementary Information:**

The online version contains supplementary material available at 10.1007/s12672-024-00998-y.

## Introduction

As a heterogeneous disease, bladder cancer accounts for approximately 573 000 new cases and 213 000 deaths per year, and it is the 6th most common cancer and the 9th leading cause of cancer death worldwide [1]. Current care options include surgery, cisplatin-based chemotherapy, radiation therapy, and immunotherapy, depending on the histology category, which is broadly classified into non-muscle-invasive bladder (NMIBC), muscle-invasive bladder cancer (MIBC), or metastatic bladder cancer [2]. However, lower initial response rates, side effects, and therapeutic resistance inevitably occur, leading to therapeutic failure or poor prognosis, particularly for those with advanced or metastatic disease [3]. Even effective biomarkers based on transcriptomic data have been established for prognostic prediction, the role of which in bladder cancer is still largely unknown, making it difficult to predict whether a certain treatment benefits bladder cancer patients [4]. Therefore, it is necessary to find reliable biomarkers to guide the clinical treatment and prognosis of bladder cancer.

Pyroptosis was discovered as a form of lytic programmed cell death triggered by inflammasomes, which sense cytosolic invasive infection or danger signals [5]. Recent documents demonstrated that pyroptosis exerted a vital role in various cancer development [6]. In hepatocellular carcinoma, pyroptosis was strongly inhibited, activation of which could significantly suppress its progression [7, 8]. The impairment of pyroptosis in triple-negative breast cancer could also remarkably promote cell proliferation potential through mitochondrial uncoupling protein 1 (UCP-1) [9]. Indeed, pyroptosis induction has become one of the mechanisms for many chemotherapy drugs to reduce tumor growth [10]. It should be noted that the function of pyroptosis was closely associated with cytotoxic lymphocytes such as natural-killer and CD8 + T lymphocytes, which indicates the process of pyroptosis is closely associated with the immune microenvironment [11]. Hence, the alteration of pyroptosis might be linked to the anti-tumor effect and the immune responses of cancer patients after immunotherapy.

Long noncoding RNAs (lncRNAs) point to transcripts of more than 200 nucleotides that lack translational activities [12]. The lncRNAs have received widespread attention currently regarding their multiple functions in biological processes, including cell-cycle regulation, the establishment of cell identity, and pyroptosis [13, 14]. Additionally, accumulated evidence demonstrates that aberrant expression of lncRNAs is involved in tumor development [15]. Moreover, the lncRNA has been proposed as relevant for cancer immunity regulation and tumor microenvironment, and many lncRNAs have been utilized as prognostic biomarkers for several types of cancers, such as kidney cancer, hepatocellular cancer, and lung cancer [16–19]. Therefore, lncRNAs might be of significant clinical relevance and useful biomarkers for survival outcomes prediction of bladder cancer. In the present study, a pyroptosis- and immune-related lncRNA signature was constructed to evaluate the prognosis prediction and the response to clinical treatment in patients with bladder cancer.

## Materials and methods

### Transcriptional and clinical information on bladder cancer acquisition

RNA-seq data of 412 tumor tissue samples and 19 paracancerous (normal) tissue samples of bladder cancer were retrieved from the TCGA database (https://tcgadata.nci.nih.gov/tcga/). We obtained 412 clinical data of bladder cancer patients from TCGA. The patients with missing clinical features or overall survival (OS) of less than 30 days were excluded. The Supplementary Table 1 showed all the clinical characteristics of bladder cancer patients.

### Acquisition of pyroptosis- and immune-related lncRNAs

A total of 404 pyroptosis-related genes were retrieved from GeneCards (https://www.genecards.org/). From the ImmPort database (http://www.immport.org), a total of 2484 immune-related genes were downloaded. We obtained differentially expressed genes through the limma R package (thresholds of |log fold change (FC)|> 1 and false discovery rate (FDR) < 0.05) (version 3.53.10), respectively. The correlation between pyroptosis/immune-related genes and lncRNAs were calculated by limma R package. We identified the pyroptosis-related lncRNAs (PyrolncRNAs) and immune-related lncRNAs (ImmlncRNAs) by using the screening criteria (|correlation coefficient|> 0.3, p-value < 0.01). Pyroptosis- and immune-related lncRNAs (Pyro-Imm lncRNAs) were revealed by calculating the intersection of differentially expressed PyrolncRNAs and ImmlncRNAs with the Venn diagram (http://bioinformatics.psb.ugent.be/webtools/Venn/).

### Creation of pyroptosis- and immune-related lncRNA prognostic model

We used univariate Cox regression analysis to obtain the Pyro-Imm lncRNAs associated with prognosis (p < 0.05). After least absolute shrinkage and selection operator (LASSO) regression analysis, multivariate Cox regression analysis was carried out to screen the suitable Pyro-Imm lncRNAs to construct a predictive signature (p < 0.05). The risk score was calculated as follows: risk score = Ʃ[Coef(lncRNA) × Exp(lncRNA)]. Coef (lncRNA) and Exp(lncRNA) represent the regression coefficient of the multivariate Cox analysis for the Pyro-Imm lncRNAs and Pyro-Imm lncRNAs expression value, respectively. All analyses were performed using the “survival,” “glmnet,” “survminer,” “caret,” “pheatmap,” “ggplot2”, “ggalluvial”, “dplyr,” and “survivalROC” R packages. Cytoscape software was used to visualize the network between lncRNA and mRNA.

### Validation of pyroptosis- and immune-related lncRNAs prognostic risk model

To examine the difference in survival between the low- and high-risk groups, we performed a time-dependent receiver operator characteristic (ROC) analysis, Kaplan–Meier analysis, risk score analysis, and survival outcome analysis. Univariate and multivariate Cox regression analysis and multi-index ROC analysis were used to demonstrate whether this model could be an independent clinical prognostic predictor. Additionally, using the chi-square test, we assessed the relationship between the risk score and the clinical characteristics. The associations between the Pyro-Imm lncRNAs and clinical traits were also evaluated. For these analyses, we applied “survivalROC” “survminer,” “pheatmap,” “timeROC,” “beeswarm”, and “survival” R packages.

### Nomogram establishment

Based on risk score and clinicopathological factors, including age, sex, tumor stage, T stage (the size and extent of the main tumor), and N stage (the number of nearby lymph nodes), a nomogram was constructed to predict the 1, 3, and 5 year survival of patients. The calibration curve was used to predict the accuracy of the established nomogram. The “rms” R package was performed for this analysis.

### Gene set enrichment analysis (GSEA)

GSEA software (version 4.2.1) was used to conduct the analysis. Kyoto Encyclopedia of Genes and Genomes (KEGG) pathway and gene ontology (GO) analysis were performed to evaluate the differential signaling pathways and biological processes as well as a molecular function between the low-risk group and the high-risk group (Nominal p-value < 0.05, FDR value < 0.05).

### Immune-infiltrated cells and immune checkpoints

The correlations between immune infiltration and risk score were analyzed using single-sample gene set enrichment analysis (ssGSEA). The relationships between immune checkpoint genes and risk score were further investigated. We employed the “GSEABase”, “GSVA”, “reshape2”, “ggplot2”, “ggpubr”, and “limma” R packages for these analyses.

### The response of predictive signature in clinical treatment

Based on the half-maximal inhibitory concentration (IC50), the difference between the low- and high-risk groups with selected drug candidates was compared (P < 0.05) using the “pRRophetic” and “ggplot2” R packages.

### Statistical analysis.

All analyses in this study were performed by R software (version 4.2.1), except for 1.8 (version 3.6.3). The Wilcoxon test was employed to compare gene expression levels between tumor and control groups. Kaplan-Meier survival curves and log-rank analysis were utilized to assess the OS between low- and high-risk groups. For all statistical tests, p < 0.05 was considered statistically significant.

## Results

### Identification of dysregulated pyroptosis- and immune-related lncRNAs

**Fig. 1:**
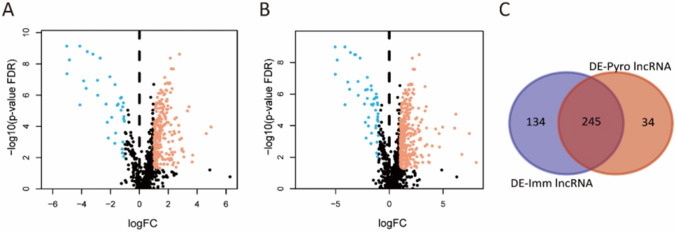
Identification of differentially expressed PyrolncRNAs (DE-Pyro lncRNAs) and immune-related lncRNAs (DE-Imm lncRNAs) in bladder cancer. **A** Volcano plot of DE-Pyro lncRNAs. Blue dots: down-regulation. Orange dots: up-regulation. **B** Volcano plot of DE-Imm lncRNAs. Blue dots: down-regulation. Orange dots: up-regulation. **C** Venn analysis between the DE-Pyro lncRNAs and DE-Imm lncRNAs

The transcriptome data downloaded from the TCGA database was divided into mRNA and lncRNA datasets. We identified 59 dysregulated pyroptosis-related genes and 316 dysregulated immune-related genes. We screened out 279 differentially expressed PyrolncRNAs (DE-Pyro lncRNAs) from 797 PyrolncRNAs (Fig. 1A) and 379 differentially expressed immune-related lncRNAs (DE-Imm lncRNAs) from 1194 immune-related lncRNAs (Fig. 1B). Then, Venn analysis between the DE-Pyro lncRNAs and DE-Imm lncRNAs was performed, generating 245 differentially expressed pyroptosis- and immune-related lncRNAs (Fig. 1C).

### Prognostic model construction

**Fig. 2:**
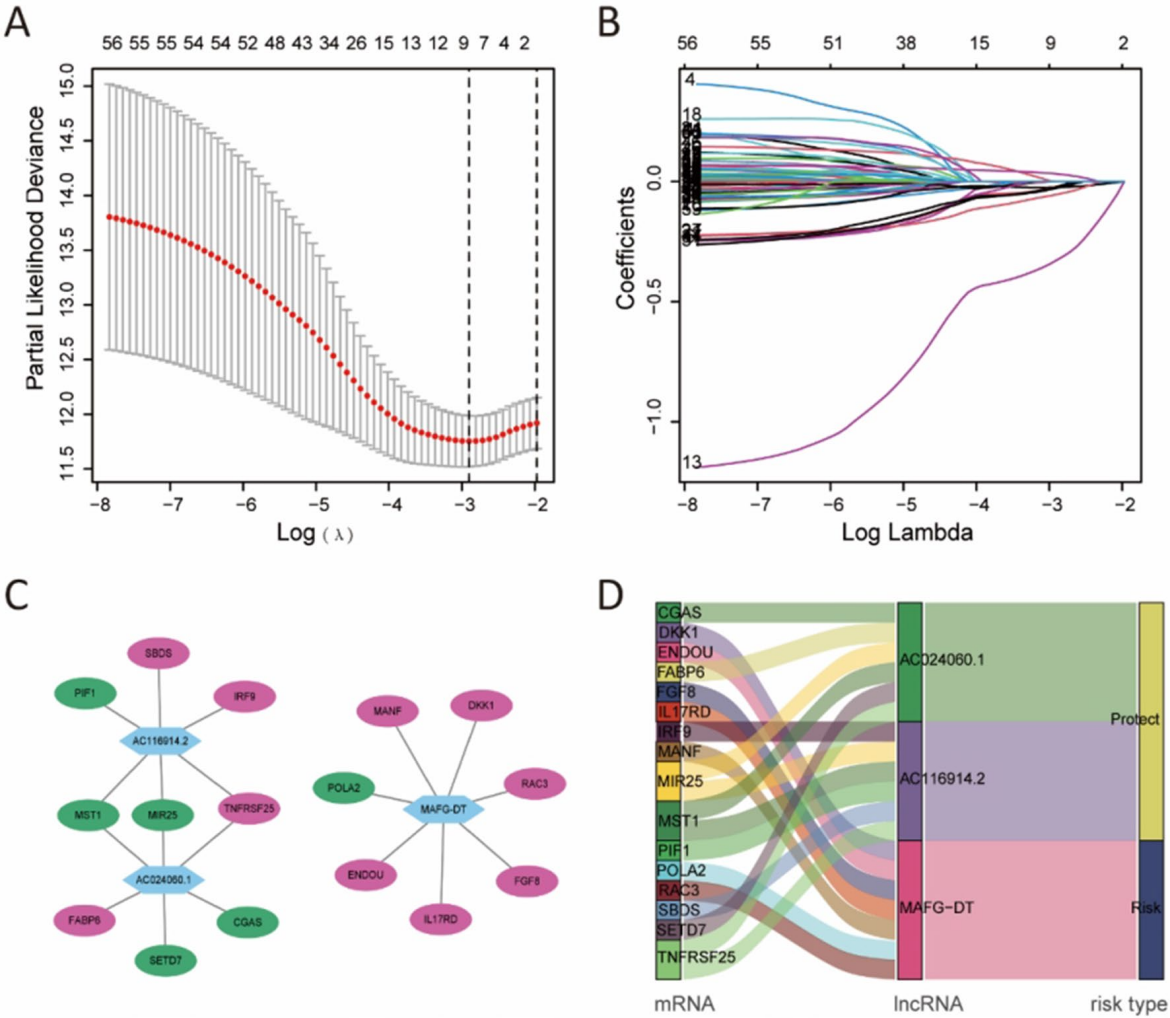
Establishment of a risk score model. **A**, **B** LASSO regression was performed based on 60 Pyro-Imm lncRNAs obtained by univariate Cox regression analysis. **C** The co-expression network of lncRNAs and mRNAs. Blue color: Pyro-Imm lncRNAs. Green color: Pyroptosis-related mRNAs. Purple color: immune-related mRNAs. **D** Sankey diagram showing the correlations between prognostic Pyro-Imm lncRNAs, mRNAs, and risk type

60 Pyro-Imm lncRNAs associated with the prognosis of bladder cancer patients through univariate Cox regression analysis were obtained (Fig. 2A). Then, LASSO regression analysis screened seven Pyro-Imm lncRNAs (Fig. 2B). Ultimately, Multivariate Cox regression revealed three Pyro-Imm lncRNAs (MAFG-DT, AC024060.1, AC116914.2) to construct a prognostic model. Risk score = (0.5185 × MAFG-DT expression value) + (− 0.3744 × AC024060.1 expression value) + (− 0.5498 × AC116914.2 expression value). The lncRNA-mRNA co-expression network was visualized in Fig. 2C. AC024060.1 and AC116914.2 were protective factors and MAFG-DT was a risk factor in the Sankey diagram (Fig. 2D).

### Evaluation of the prognostic risk model

**Fig. 3:**
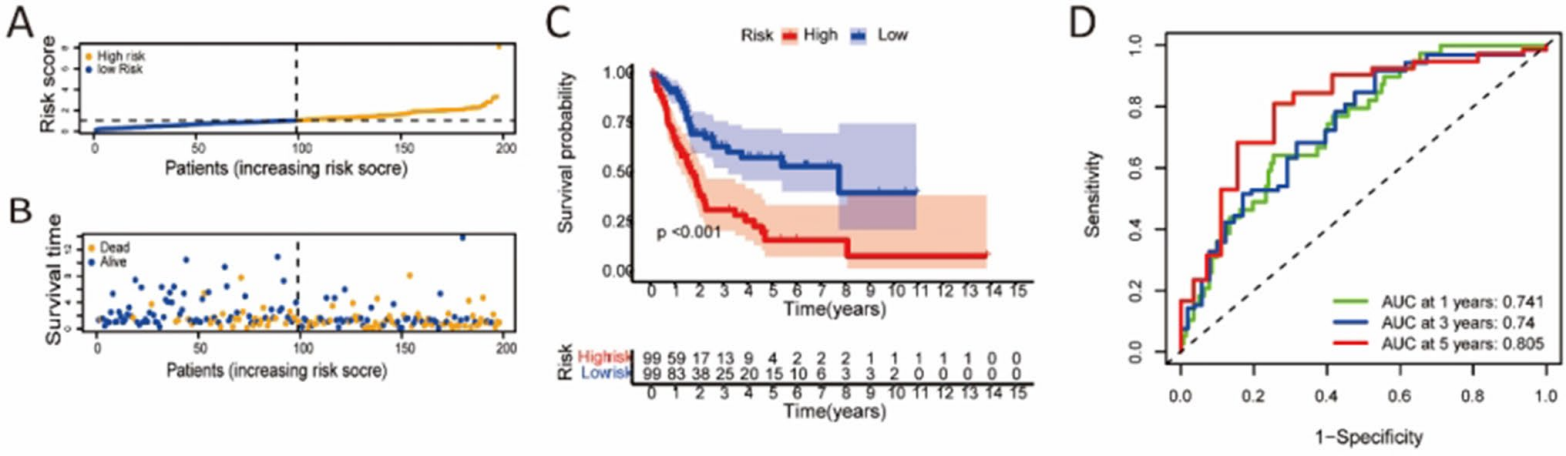
Evaluation of the risk score model. Risk scores and survival status in the first internal cohort (**A**, **B**). Kaplan-Meier tests in the first internal cohort (**C**). Time-dependent ROC analysis of risk score at 1, 3, and 5 years in the first internal cohort (**D**). AUC area is under the curve

To evaluate the predictive signature, patients were randomly separated into two cohorts (first internal or second internal cohort) and divided into low- and high-risk groups based on their median risk score. Regardless of internal cohorts or overall dataset, Kaplan-Meier analysis revealed that the OS rate in the low-risk group was significantly higher than that in the high-risk group. Here, we take the result of the first internal cohort as a representative, and the result of the second internal cohort or overall cohort was presented in the Supplementary figure 1. The risk score for the low-and high-risk groups in the first internal is shown in Fig. 3A respectively. Figure 3B displayed the survival stats of these cases. The Kaplan–Meier analysis revealed that the OS rate in the low-risk group was significantly higher than that in the high-risk group (Fig. 3C). In the time-dependent ROC curve, the first internal cohort’s area under the curve (AUC) at 1, 3, and 5 years was 0.741, 0.74, and 0.805 (Fig. 3 D).

### Evaluation of the risk signature as an independent prognostic factor for bladder cancer

The HRs (95% CI) of risk score were 1.521 (1.328–1.742) in the univariate Cox regression analysis (p-value < 0.001, Supplementary figure 2 A) and 1.380 (1.196–1.593) in the multivariate Cox regression analysis (p-value < 0.001, Supplementary figure 2 A, B). Multi-index ROC analysis showed that the AUC of risk score was 0.731, higher than the clinicopathological characteristics (age, sex, tumor stage, T stage, and N stage) (Supplementary figure 2 C). Additionally, principal component analyses (PCA) showed that patients with different risk scores could be better distinguished based on the three Pyro-Imm lncRNAs (Supplementary figure 2 F), compared with the whole genes (Supplementary figure 2 D) and the overlapping 245 Pyro-Imm lncRNAs (Supplementary figure 2 E).

### The risk model was associated with prognosis in different clinicopathological features

Bladder cancer patients were stratified by the clinicopathologic features, including age, sex, tumor stage, grade, T stage, and N stage. According to Kaplan-Meier survival analysis, the age, male, high grade, tumor stage III-IV, T stage, and N stage were associated with higher survival probability in the low-risk group compared with that in the high-risk group (Supplementary figure 3 A–I).

### Relationships between Pyro-Imm lncRNAs and clinicopathological features and nomogram construction

In the risk prognosis model, the 3 Pyro-Imm lncRNAs were related to clinicopathological features. MAFG-DT was associated with survival status, age, and N stage (Supplementary figure 4 A, B, C). AC024060.1 was related to survival status and grade (Supplementary figure 4 D, E). AC116914.2 was connected to the survival status, grade, tumor, T, and N stages (Supplementary figure 4 F–J). Besides, containing the risk score and clinicopathological factors, the nomogram could predict the 1, 3, and 5 year prognosis of bladder cancer patients (Supplementary figure 5, A). The predicted survival rates were consistent with the actual OS rates at the time of 1, 3, and 5 year, demonstrating the nomogram’s strong predictive ability (Supplementary figure 5 B).

### Gene set enrichment analysis

**Fig. 4:**
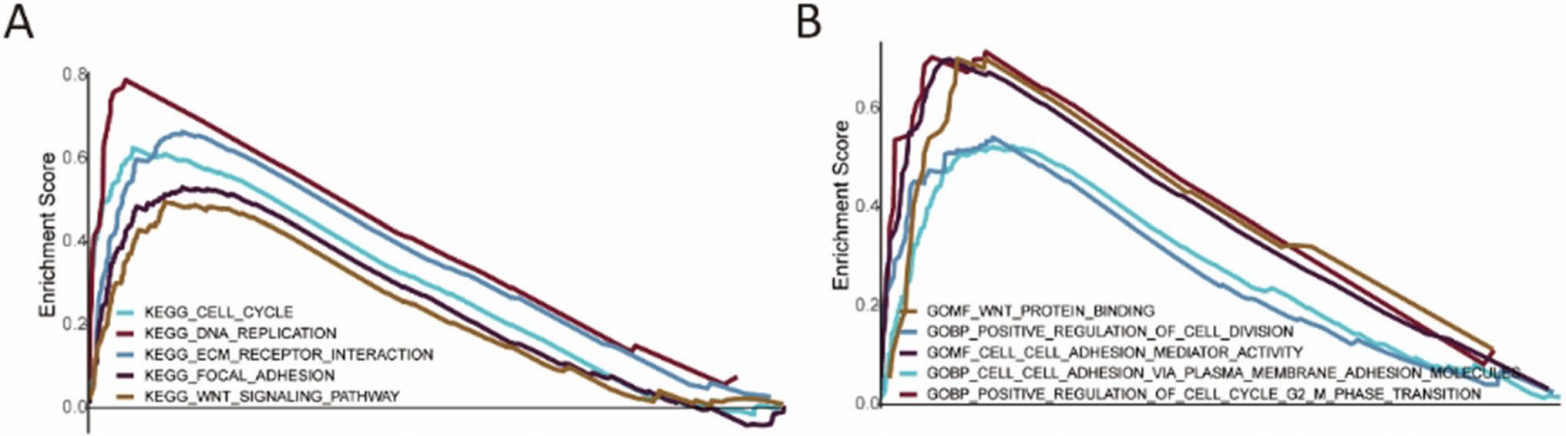
Gene Set Enrichment Analysis. **A** KEGG pathway analysis showed five pathways were enriched in the high-risk group. **B** Five GO items were enriched in the high-risk group. (ECM represents extracellular matrix)

GSEA revealed that KEGG pathways, including the WNT signaling pathway, cell cycle, DNA replication, focal adhesion, and ECM (extracellular matrix) receptor interaction were significantly enriched in the high-risk group, while no signaling pathways were enriched in the low-risk group (Fig. 4 A). The GO analysis showed that the high-risk group enriched in cell adhesion via plasma membrane adhesion molecules, cell adhesion mediator activity, positive regulation of cell cycle G2/M phase transition, positive regulation of cell division, and WNT protein binding, whereas no items were enriched in the low-risk group (Fig. 4 B).

### Immune infiltration and immune checkpoints

**Fig. 5:**
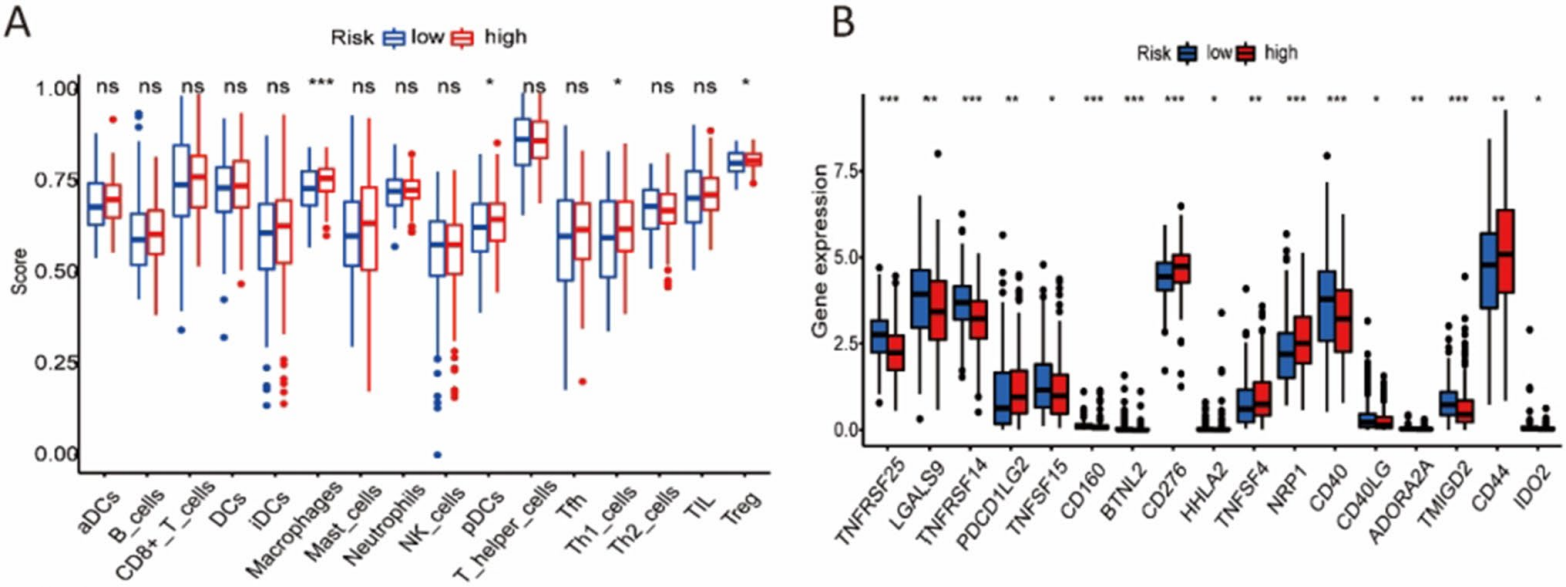
Immune infiltration and immune checkpoints. **A** The infiltration levels of 16 immune cells in the low-risk and high-risk groups. **B** The expression value of 17 immune checkpoints in the low-risk and high-risk groups. *p < 0.05, **p < 0.01, ***p < 0.001, ns: not significant

We investigated the correlations between immune cells and risk score. The results showed that macrophages, plasmacytoid dendritic cells (pDCs), T helper type 1 cells (Th1), and regulatory T cells (Treg) were significantly higher in the high-risk group, compared with the low-risk group (Fig. 5 A). We further evaluated the relationships between the expression value of immune checkpoint genes and the two risk groups. The immune checkpoint genes, including TNFRSF14, TNFRSF15, TNFRSF25, LGALS9, BTNL2, HHLA2, CD40, CD40LG, CD160, ADORA2A, TMIGD2, and IDO2 were highly expressed in low-risk group, while TNFRSF4, PDCD1LG2, NRP1, CD44, and CD276 were substantially expressed in high-risk group (Fig. 5 B). However, the expression of PDL-1 and CTLA4 was no difference between high-risk and low-risk group. The results indicate that the two risk groups' immune responses varied and may react differently to immunotherapy.

### Relationship between the risk model and bladder cancer treatment

**Fig. 6:**
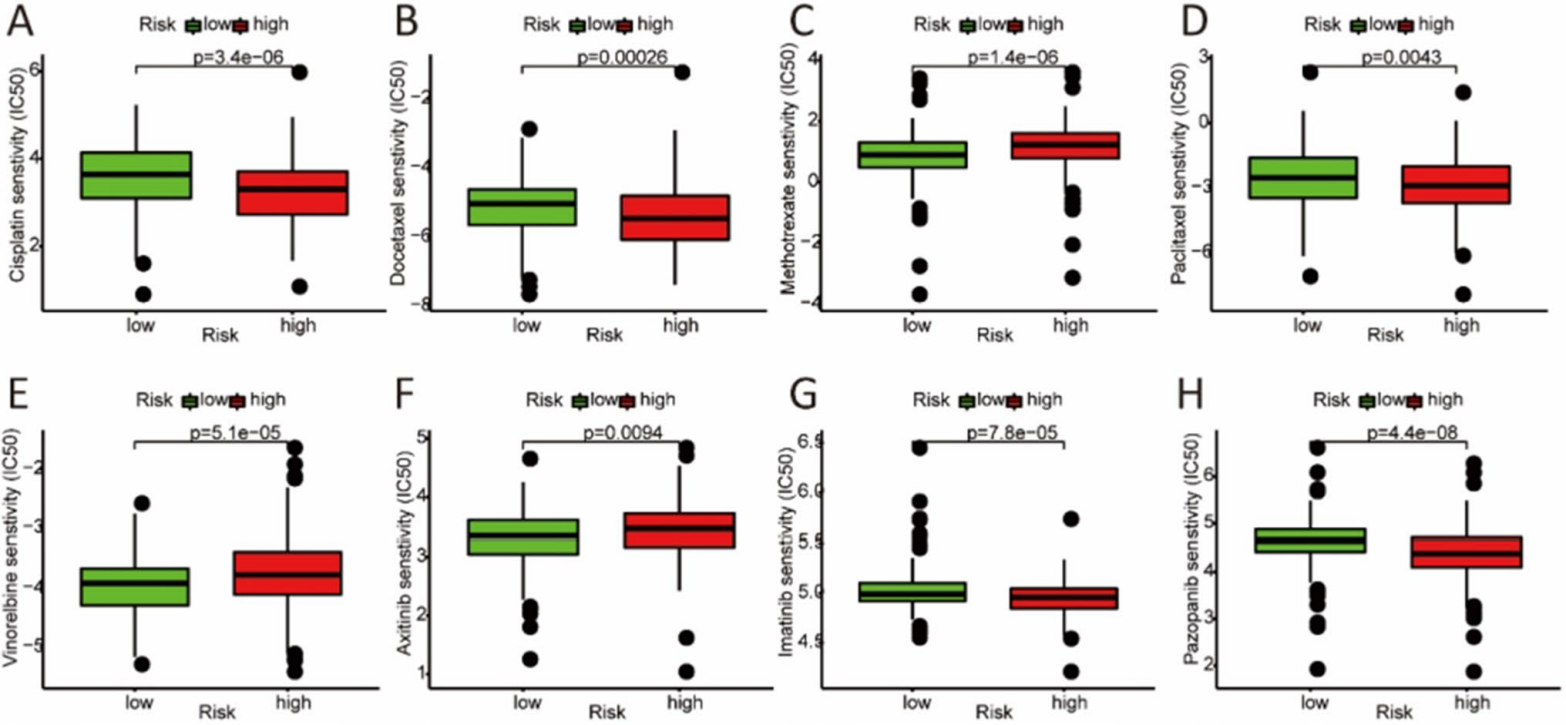
The efficacy of bladder cancer treatment in low-risk and high-risk groups. The IC50 of cisplatin **A**, docetaxel **B**, methotrexate **C**, paclitaxel **D**, vinorelbine **E**, axitinib **F**, imatinib **G**, and pazopanib **H** were compared between low-risk and high-risk groups. (IC50 represents half-maximal inhibitory concentration)

We analyzed the relationship between the risk model and the efficacy of general chemotherapeutic and targeted drug treatment for bladder cancer. The results showed that the IC50 values of cisplatin, docetaxel, paclitaxel, imatinib, and pazopanib were lower in the high-risk group, whereas the IC50 values of methotrexate, vinorelbine, and axitinib were higher in the high-risk group (Fig. 6).

## Discussion

Given several PyrolncRNAs have been found for the potential bladder cancer prognosis biomarkers, there is a paucity of data on the combination of ImmlncRNAs and PyrolncRNAs in bladder cancer prognosis analysis [4, 12]. Based on public databases (TCGA, GeneCards, and ImmPort), we integrated ImmlncRNAs into PyrolncRNAs research to achieve a novel and more comprehensive risk model for prognosis prediction. In the current study, three Pyro-Imm lncRNAs including MAFG-DT, AC024060.1, and AC116914.2 have been found and used for the bladder cancer risk model construction. Previous evidence showed that the MAFG-DT might be an indicator of infiltration of mononuclear immune cells and linked to the immunosuppressive phenotype of bladder cancer, which may explain why the MAFG-DT is a risk factor in our data [16]. The AC024060.1 was considered a protective factor for bladder cancer patients in our study, which is consistent with the analysis from other authors [20]. Regarding AC116914.2, no data on bladder cancer has been found yet. However, it has been applied as a risk model in clear cell renal cell carcinoma to predict survival status and tumor immune infiltration [21].

The risk score from the three Pyro-Imm lncRNAs model is an independent and better predictor for the prognosis of bladder cancer patients in comparison to other clinicopathological characteristics such as age, sex, tumor stage, T stage, and N stage. Patients being divided into a low-risk group according to the median risk score have higher overall survival rates than the patients in a high-risk group. Our analysis also shows that Treg cells have higher infiltration in the high-risk group relative to the low-risk group, and the high infiltration levels of Treg cells are generally associated with poor prognosis [22]. These results indicate that grouping due to the risk score is highly clinical relevance since urologists may use it to distinguish different risk patients and assist the decision-making of individualized drug medicine for more precise therapy.

Pyroptosis is closely involved in immune cell infiltration. Tumor-infiltrating immune cells can trigger pyroptosis, affecting the prognosis of bladder cancer patients [22]. Indeed, increased CD11b + cells infiltrating in bladder cancer 5637 cells microenvironment delayed tumor growth, which is attributed to inflammasome activation and elevated level pyroptosis [23]. lncRNA signature of tumor-infiltrating B lymphocytes was identified to predict the prognosis and response to immunotherapy of bladder cancer [16]. Nevertheless, the role of pyroptosis in bladder cancer is not clear yet. GSDMB, a member of the gasdermin protein family participating in the provoking of pyroptosis, has been proven to promote the progression of bladder cancer by activating the signal transducer and activator of transcription 3 (STAT3) [24]. The controversial roles of pyroptosis are hard to explain. Oltra and co-authors demonstrated that GSDMB-mediated pyroptosis is detrimental to GSDMB cleavage, uncleaved of which promotes pro-tumor effects. Meanwhile, various GSDMB isoforms cleaved by specific proteases have different effects on pyroptosis regulation, and only GSDMB isoforms containing exon 6 translation can induce cancer cell pyroptosis [25]. It indicates that the successful induction of pyroptosis might be a key step for its anti-tumor function. Further investigations are also required for a definitive answer on whether pyroptosis or to what degree pyroptosis is beneficial for bladder cancer treatment.

Both KEGG and GO enriched analysis revealed that the WNT signaling pathway emerged in the high-risk group based on the risk model, which means the aberrant WNT pathway is one of the probable mechanisms involved in a worse survival rate. Evidence shows that aberrant-activated this pathway could enhance bladder cancer cell proliferation, invasion, migration, and epithelial-to-mesenchymal transition (EMT) [26]. In contrast, inhibition of the canonical WNT/β-catenin pathway reversed the EMT process and suppressed the invasion and metastasis of bladder cancer cells [27]. Targeting this pathway, therefore, might be a promising therapeutic choice for a better survival outcome. However, these in vitro findings have not been verified by patient studies. Pertinent clinical trials about the therapeutic role of the WNT/β-catenin pathway inhibitor in bladder cancer patients should be further performed.

It is worth noting that lncRNAs can participate in the development of bladder cancer through the WNT/β-catenin pathway. By activating this signaling pathway, lncRNA SNHG20 significantly promotes the malignant progression of bladder cancer [28]. Another lncRNA PVT1 has been proven to accelerate malignant phenotypes of bladder cancer cells via the overactivation of the WNT/β-catenin pathway [29]. Importantly, lncRNAs are also involved in the development of chemoresistance via regulation of the WNT/β-catenin signaling pathway. Overexpressed lncRNA NEAT1 results in cisplatin-resistance development in bladder cancer T24 cell line through WNT pathway activation [30]. lncRNA UCA1 increases the chemoresistance of cisplatin in bladder cancer treatment by activating the WNT signaling [31]. In addition, the lncRNA CDKN2B antisense RNA 1 gene can inhibit gemcitabine sensitivity via the induction of WNT signaling in bladder urothelial carcinoma [32]. It is not surprising that the high-risk group of our risk model is more sensitive to cisplatin (with a lower IC50 value), whereas the low-risk group is more sensitive to methotrexate (with a lower IC50 value). This suggests that urologists might give sensitive chemotherapeutic drugs to different patients based on our risk model. It is postulated that cisplatin plus gemcitabine combination therapy might be beneficial for the high-risk group, while the Methotrexate, Vinblastine, Doxorubicin, and Cisplatin (MAVC) regime may be beneficial for the low-risk group. However, this is purely speculated, and whether this postulation is clinically relevant needs further validation.

With a greater understanding of molecular mechanisms, immunotherapy has revolutionized the approach to urothelial carcinoma. For example, immune checkpoint inhibitors targeting PD-1 or PD-L1 have been approved by the Federal Food and Drug Administration (FDA) for patients who progressed after cisplatin-based chemotherapy or were ineligible for cisplatin-based chemotherapy [33]. Nevertheless, the objective response rates for advanced bladder cancer patients only range from 15 to 31% [34]. This is probably because these unresponsive patients have no corresponding immune checkpoint gene expression limiting the application of immunotherapies. Identifying the expression of immune checkpoint genes in advanced cancer patients before immunotherapy is of great importance in improving immunotherapeutic efficiency. Our study reveals that the expression of immune checkpoint genes was significantly different between the high-risk and low-risk groups. The utilization of our risk model may offer useful information for individualized and precise immunotherapy in the future.

Several limitations of our study should not be ignored. Our research data is based on public datasets, which might be limited due to insufficient sample size for certain analyses. Since we did not verify the reliability of our Pyro-Imm lncRNAs signature in another independent cohort, potential errors or deviation may inevitably occur. Moreover, in vitro, in vivo, or clinical studies are still lacking for further validating the practical use of this risk model.

### Supplementary Information

Additional file1 (DOCX 1459 KB)

## Data Availability

The datasets generated during the current study are available in the in TCGA database (https://tcgadata.nci.nih.gov/tcga/).
