# Supplementary material for: Identification of a pyroptosis-immune-related lncRNA signature for prognostic and immune landscape prediction in bladder cancer patients
Source: Discov Oncol. 2024 May 2;15:140. doi: 10.1007/s12672-024-00998-y (PMC11065857; doi:10.1007/s12672-024-00998-y)
Supplement: Supplementary file 1 — Additional file1 (DOCX 1459 KB) [file 12672_2024_998_MOESM1_ESM.docx]

**Identification of a pyroptosis-immune-related lncRNA signature for prognostic and immune landscape prediction in bladder cancer patients**

Journal of Cancer Research and Clinical Oncology

Author: Fuguang Zhao and Zhibo Jia

Corresponding author: Hui Xie, Departments of Urology, the First Affiliated Hospital of Fujian Medical University, Fuzhou 350005, P.R. China. Email: xiehui0831@outlook.com

| **Characteristic** | **Variable** | **Total (n = 412)** | **%** |
| --- | --- | --- | --- |
| Age(years) | ≤ 65 | 162 | 39.32 |
|  | > 65 | 250 | 60.68 |
| Gender | Male | 304 | 73.79 |
|  | Female | 108 | 26.21 |
| Grade | High grade | 388 | 94.17 |
|  | Low grade | 21 | 5.10 |
|  | Unknown | 3 | 0.73 |
| Stage | I | 2 | 0.49 |
|  | II | 131 | 31.80 |
|  | III | 141 | 34.22 |
|  | IV | 136 | 33.01 |
|  | Unknown | 2 | 0.49 |
| Tumor classification | T0 | 1 | 0.24 |
|  | T1 | 3 | 0.73 |
|  | T2 | 120 | 29.13 |
|  | T3 | 196 | 47.57 |
|  | T4 | 59 | 14.32 |
|  | TX | 1 | 0.24 |
|  | Unknown | 32 | 7.77 |
| Lymph nodes | N0 | 239 | 58.01 |
|  | N1 | 47 | 11.41 |
|  | N2 | 76 | 18.45 |
|  | N3 | 8 | 1.94 |
|  | NX | 36 | 8.74 |
|  | Unknown | 6 | 1.46 |
| Distant metastasis | M0 | 196 | 47.57 |
|  | M1 | 11 | 2.67 |
|  | MX | 202 | 49.03 |
|  | Unknown | 3 | 0.73 |
| Survival status | Alive | 253 | 61.41 |
|  | Death | 159 | 38.59 |

**Supplementary table 1.** All the clinical characteristics of bladder cancer patients.


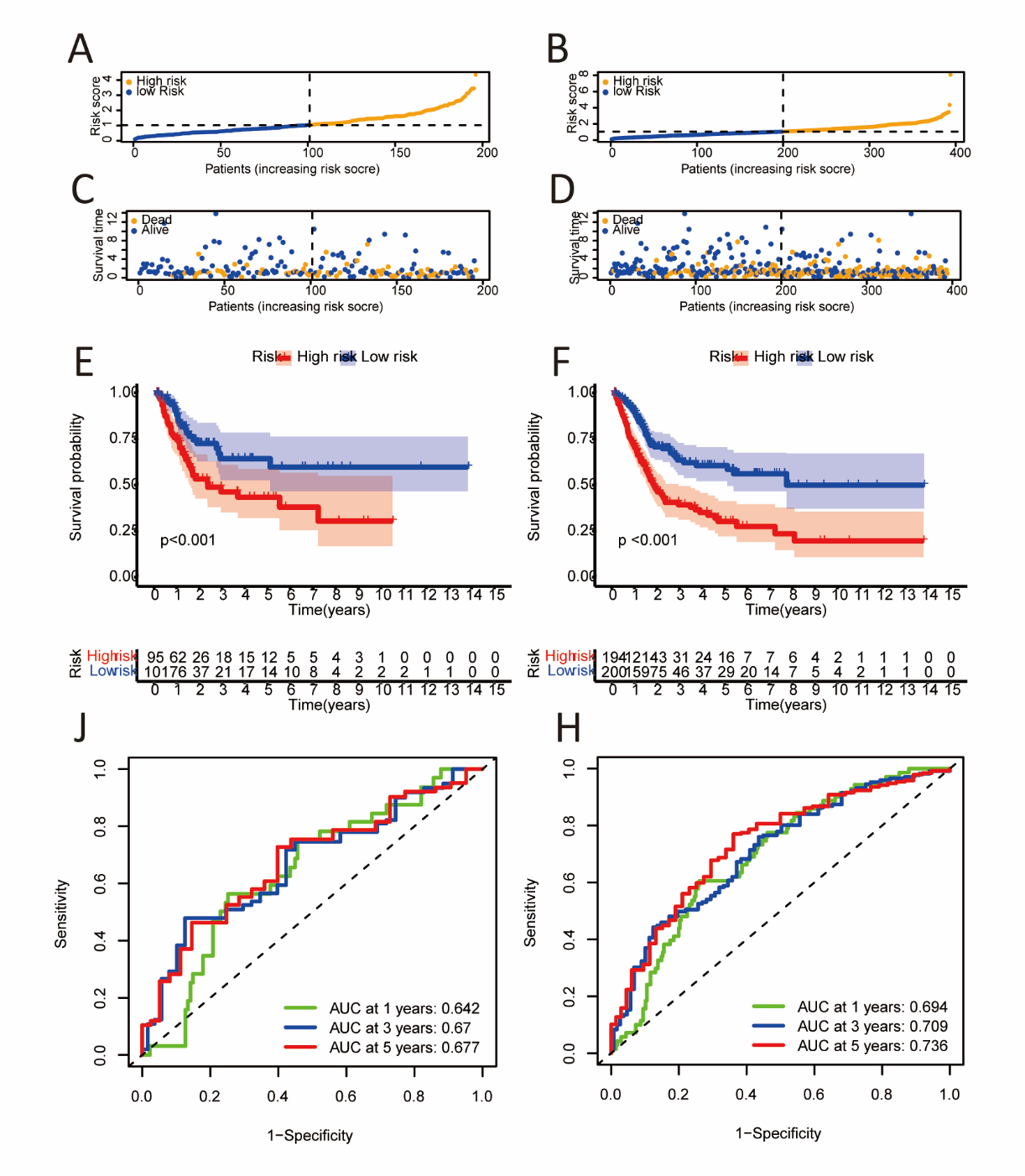


**Supplementary figure 1.** Evaluation of the risk score model. Risk scores and survival status in second internal cohort (A, C) and entire cohort (B, D). Kaplan–Meier tests in first second internal cohort (E), and entire cohort (F). Time-dependent ROC analysis of risk score at 1, 3, and 5 years in second internal cohort (J), and entire cohort (H).


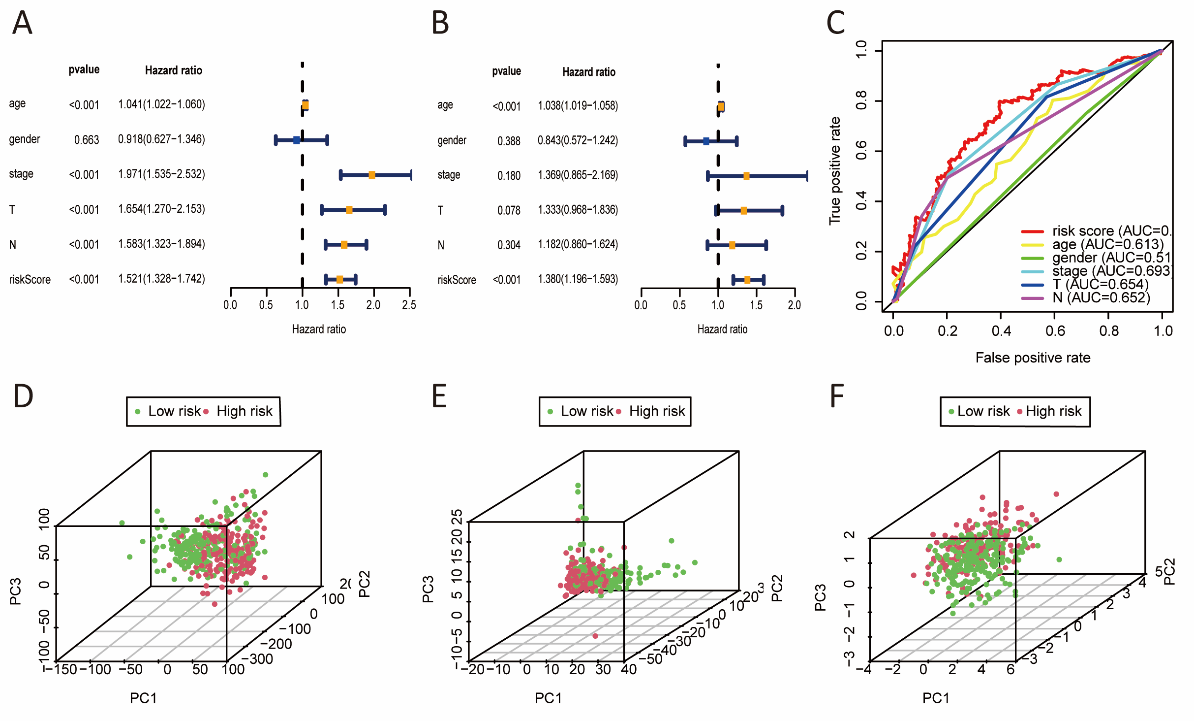
 **Supplementary figure 2.** The risk score was an independent prognostic factor of OS and principal component analyses. Univariate (A) and multivariate (B) Cox regression analysis showed the relationships between risk score and clinicopathological characteristics and OS in the entire cohort. (C) Time-dependent ROC analysis for predicting OS by prognostic factors in the entire cohort. (D) whole genes. (E) The overlapping 245 Pyro-Imm lncRNAs. (F) The risk model including three Pyro-Imm lncRNAs. (AUC, area is under the curve; T represents the size and extent of the main tumor; N represents the number of nearby lymph nodes; PC represents principal component).


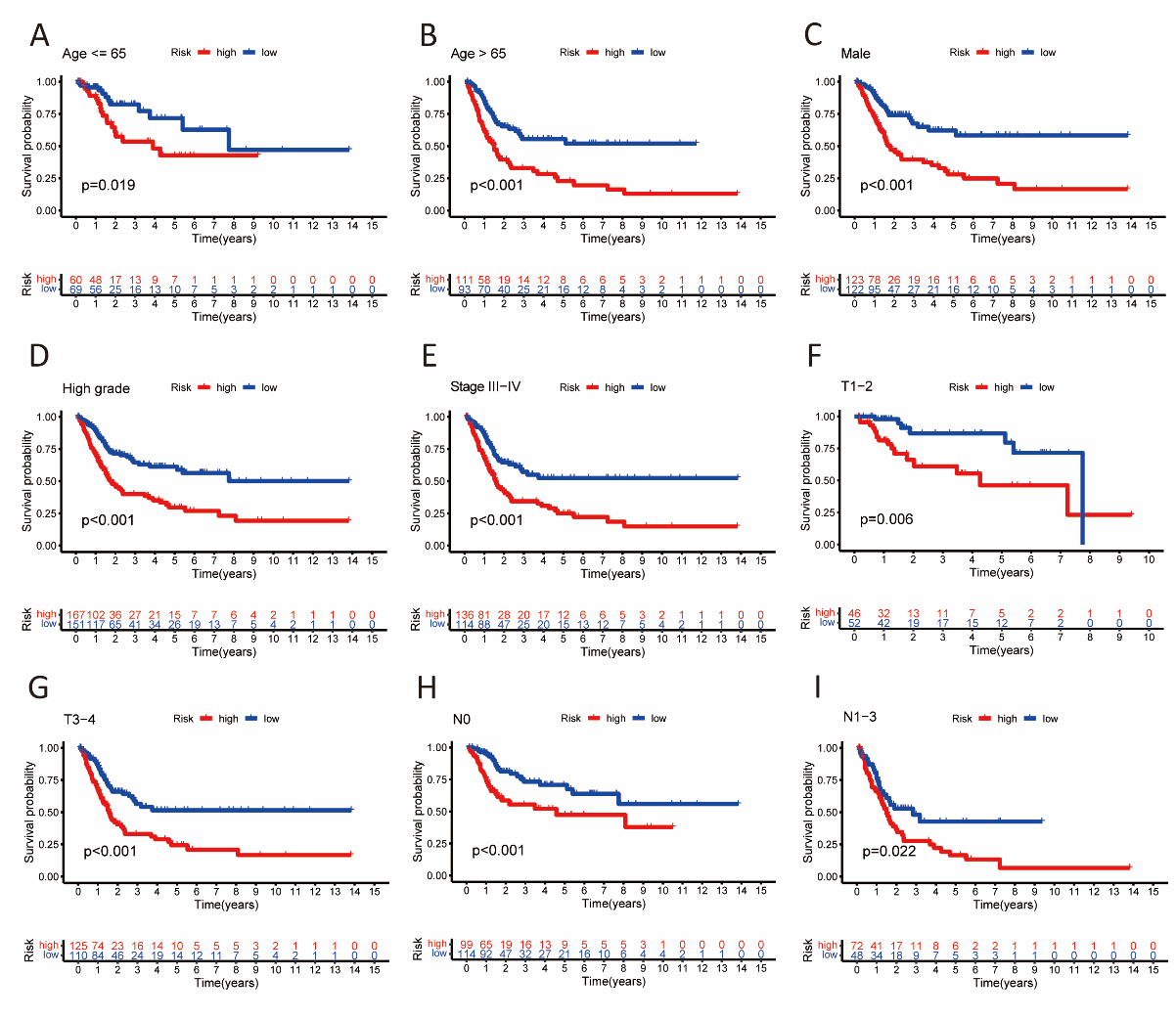
 **Supplementary figure 3.** Kaplan–Meier survival analysis in different stratified clinicopathological features. (A, B) Age. (C) Sex. (D) Grade. (E) Tumor stage. (F, G) T stage. (H, I) N stage. (T represents the size and extent of the main tumor; N represents the number of nearby lymph nodes).

**
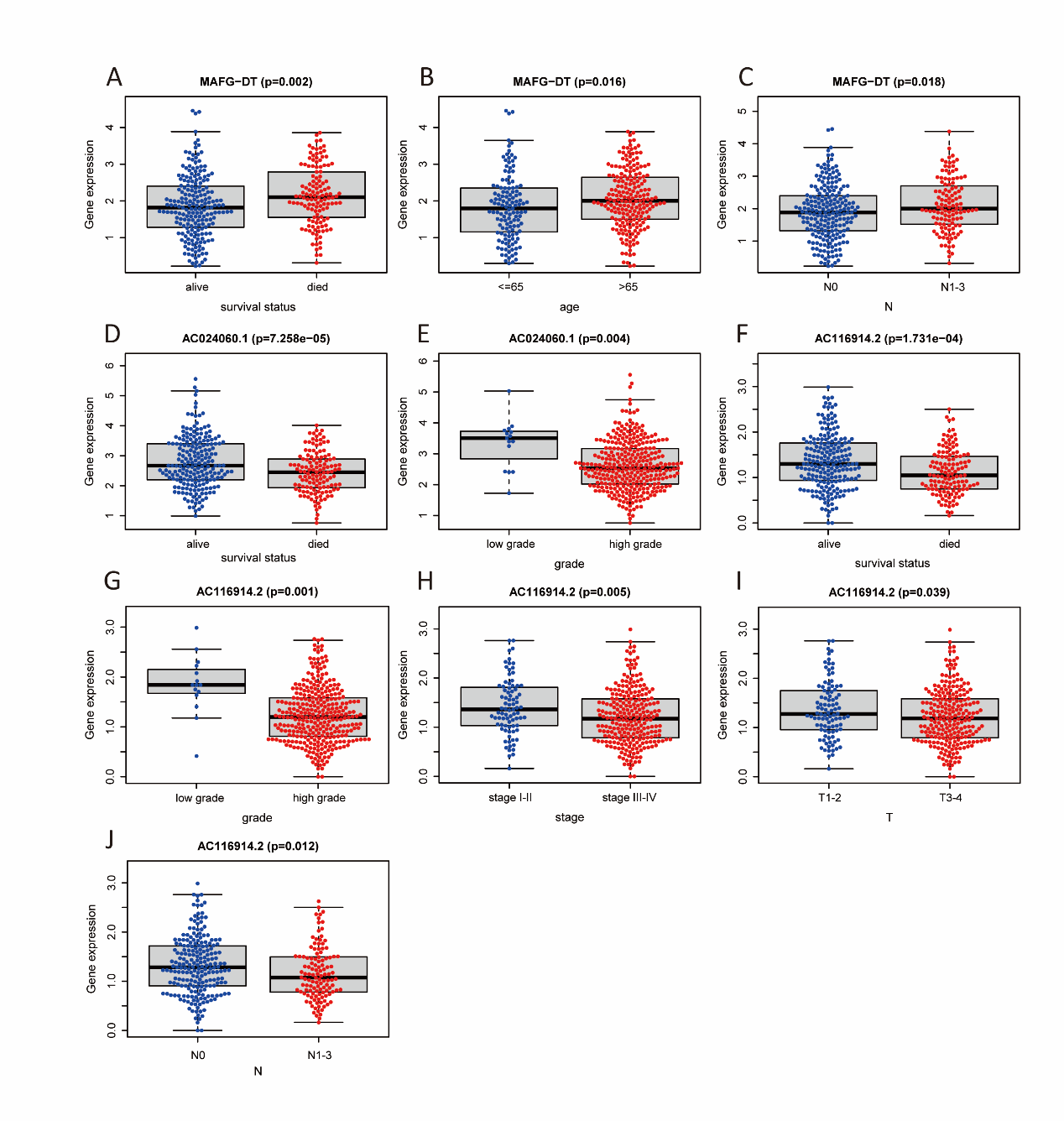
** **Supplementary figure 4.** The Relationships between the three Pyro-Imm lncRNAs and clinicopathological features. (A-C) MAFG-DT (D, E) AC024060.1 (F-J) AC116914.2. (T represents the size and extent of the main tumor; N represents the number of nearby lymph nodes).


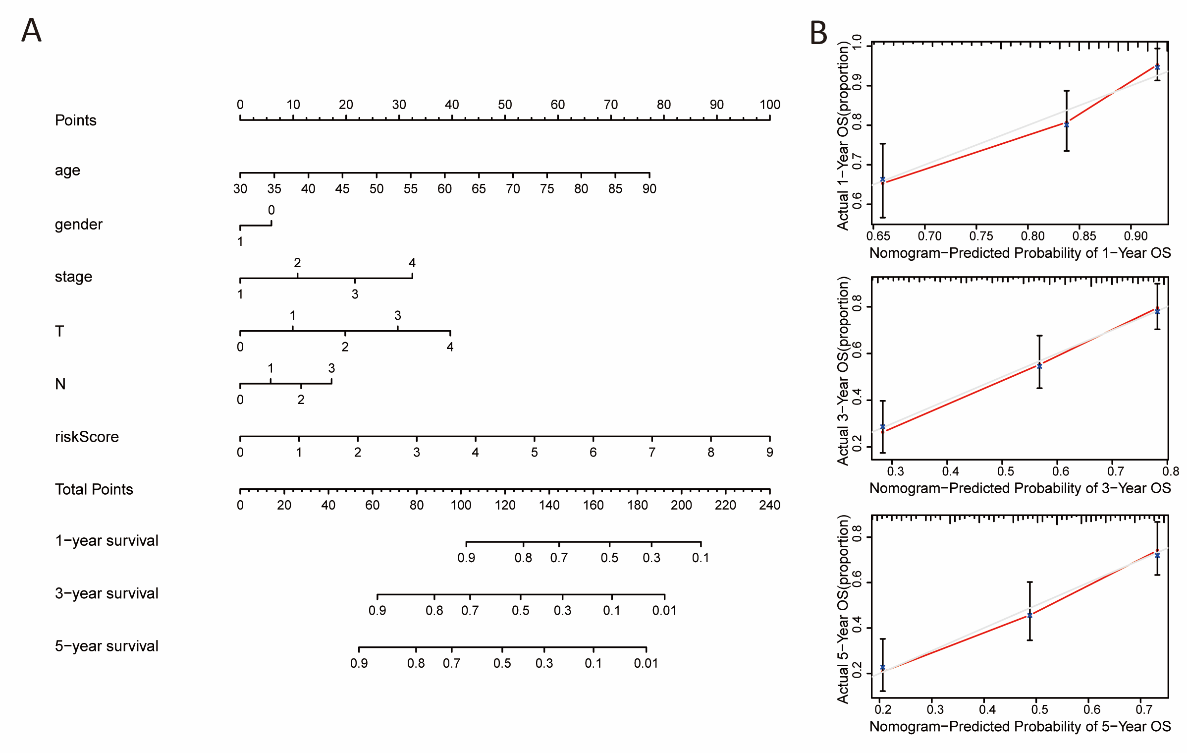
 **Supplementary figure 5.** Nomogram construction and validation. (A) Nomogram predicting 1-year, 3-year, and 5-year prognosis based on risk score and clinicopathological features. (B) Nomogram-predicted probability of 1-year, 3-year, and 5-year survival. (T represents the size and extent of the main tumor; N represents the number of nearby lymph nodes).
